# Study on mechanism of iridoid glycosides derivatives from *Fructus Gardeniae* in treatment of hepatic encephalopathy by network pharmacology and molecular docking technology

**DOI:** 10.1097/MD.0000000000041089

**Published:** 2025-01-03

**Authors:** Fangzhou Liu, Meng Li, Yuanbai Li, Yu Du, Yihao Li, Yang Yang

**Affiliations:** a Institute of Information on Traditional Chinese Medicine, China Academy of Chinese Medical Sciences, Beijing, China.

**Keywords:** *Fructus Gardeniae*, hepatic encephalopathy, iridoid glycosides, molecular docking, network pharmacology

## Abstract

**Background:**

This study aims to explore the mechanism of the iridoid glycosides from *Fructus Gardeniae* (IGFG) in treating hepatic encephalopathy (HE) by combining network pharmacology and molecular docking technology.

**Methods:**

Firstly, we collected the targets of IGFG and HE. The targets of IGFG were predicted through the CTD, SWISS and TCMSP database and the targets of HE were screened through the DisGeNET database. Then the targets of IGFG and HE were mapped to attain the common target of IGFG in treating HE. Then, chemicals-target-disease network was constructed. Secondly, we constructed protein–protein interactions (PPI) network using STRING database and Cytoscape software. Moreover, we screened the core targets according to the degree value. Thirdly, the mechanism of IGFG in treating HE was revealed by Gene ontology and KEGG enrichment analysis. Meanwhile, chemicals-target-pathway network was constructed. Finally, to further verify the analysis results, molecular docking study was conducted.

**Results:**

Network pharmacology indicates that there are 12 common targets between IGFG and HE. Eleven core targets were identified by the construction of PPI network. Association for core targets, and related pathways was analyzed, implying that core targets related to these pathways are AKT1, tumor necrosis factor, MTOR, CHUK, PPP2CA, IKBKB, AKT2, IKBKG, IL1B, NFKBIA, and CASP8. The main mechanism of IGFG in treating HE is closely related to inhibit inflammatory reaction, regulate immunity, promote hepatocyte regeneration, reduce hepatocyte apoptosis, maintain liver function homeostasis and antiviral function. Finally, the results of molecular docking showed that the binding free energy of geniposide with the core target was less than −5 kJ/mol, which indicated that geniposide could spontaneously bind to the receptor protein and had strong binding force.

**Conclusion:**

IGFG can achieve simultaneous intervention of HE by inhibit inflammatory reaction, regulate immunity, promote hepatocyte regeneration, reduce hepatocyte apoptosis, maintain liver function homeostasis and antiviral function. It presents the network regulation mechanism of mutual influence and complex correlation. This study provides a scientific basis for IGFG in the treatment of patients with HE.

## 1. Introduction

Hepatic encephalopathy (HE) is a known neurological complication of advanced liver cirrhosis^[1]^, which is a syndrome of spectrum of neuropsychiatric abnormalities caused by porto-systemic venous shunting, with intrinsic liver disease. The main clinical manifestations are the decrease of neuropsychiatric function in patients with acute or chronic liver disease,^[2]^ including mild mental state damage, behavioral disorders and coma.^[3]^ Studies have shown that 30% to 50% of hospitalized cases of cirrhosis are related to HE,^[4]^ and HE is a major independent risk factor for death in patients with liver cirrhosis.^[5]^ The pathogenesis of HE is very complex and has not been fully elucidated. At present, the classical theory of ammonia poisoning and the change of neurotransmitters have been widely accepted.^[6]^ In recent years, the theories of inflammatory reaction,^[7,8]^ intestinal flora,^[9]^ astrocyte premature aging,^[10]^ cerebral blood flow changes^[11]^ and other theories have been paid more and more attention. Pharmacological therapy of HE tend to focuses on reducing blood ammonia level.^[12]^ Clinical commonly used drugs mainly include non-absorbed disaccharide drugs such as lactulose and lactose alcohol, and nonabsorbable antibiotics such as neomycin and rifaximin.^[13]^ However, both drugs have side effects, such as electrolyte disorders, diarrhea and drug resistance.^[14]^ Therefore, it is wise to consider the therapeutic potential of herbal medicines, which are widely used in various medical fields due to their stability and multi-dimensional pharmacological effects.^[15]^

**Figure 1.**
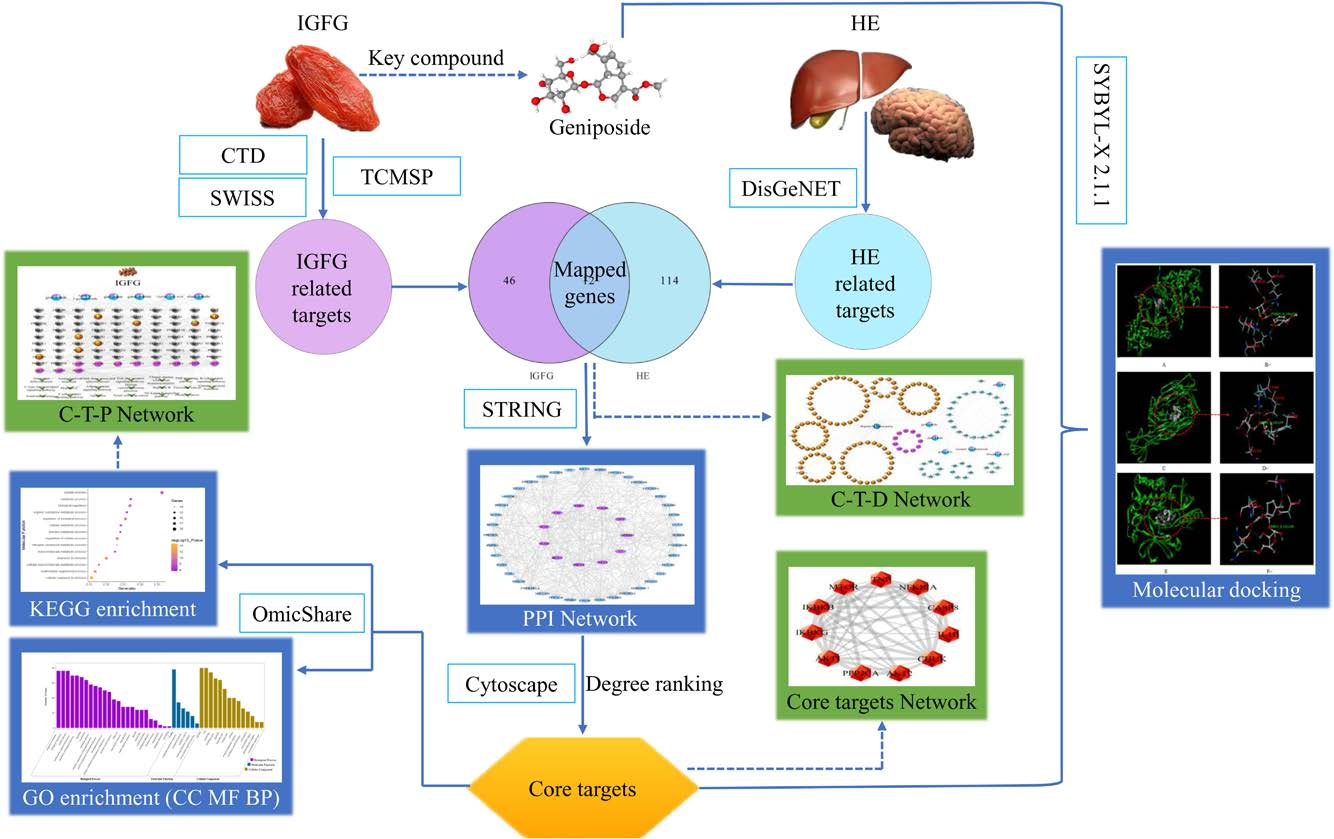
The schematic illustration of this study.

Gardeniae Fructus, the dried fruits of *Gardenia jasminoides* Ellis, is widely used as traditional Chinese medicine (TCM), which has been also contained in traditional medicine formulations for the treatment of acute or chronic hepatic diseases,^[16]^ icteric hepatitis, itching skin, eczema,^[17]^ diabetes^[18]^ and depression.^[19]^ Gardeniae Fructus is commonly applied in many Asian countries for several therapeutic proprieties and is also recorded in Chinese Pharmacopoeia.^[20]^ Modern pharmacology has demonstrated that Gardeniae Fructus has several therapeutic effects such as antiphlogistic properties, cytotoxic effects, antioxidant activity, fibrinolytic capability^[21]^ and anti-diabetes.^[22]^ Iridoid glycosides derive from Gardeniae Fructus (IGFG), including geniposide, gardenoside, shanzhiside, scandoside methyl ester, geniposidic acid and genipin gentiobioside, et al^[23]^ have been demonstrated to be the major bioactive ingredients in Gardeniae Fructus.^[24–26]^ This study aims to explore the mechanism of IGFG in treating HE using network pharmacology research methods, and to provide scientific basis for IGFG in the prevention and treatment of HE. The schematic illustration of this study is shown in Figure 1.

## 2. Materials and methods

### 2.1. Screening of potential targets for IGFG

Previous studies of our group show that Iridoid glycosides including gardenoside, geniposide, gardoside geniposidic acid, shanzhiside and genipin 1-gentiobioside from Gardeniae Fructus not only play an important role in liver diseases, but also in nervous system diseases.^[27]^ Therefore, we boldly speculate that Iridoid glycosides from Gardeniae Fructus (IGFG) can exert pharmacological effects in HE through comprehensive regulation of signaling pathways. The major bioactive ingredients of IGFG is shown in Table 1. This study comprehensively utilizes the CTD (http://ctdbase.org/),^[28]^ SWISS (http://www.swisstargetprediction.ch/)^[29]^ and TCMSP (https://old.tcmsp-e.com/tcmsp.php) database to identify potential targets associated with IGFG.

**Table 1:** The major bioactive ingredients of IGFG.

Compound	PubChem CID	MW (g/mol)	CAS
Geniposide	107,848	388.4	24512-63-8
Gardenoside	24,721,095	404.4	24512-62-7
Geniposidic acid	443,354	374.34	27741-01-1
Genipin 1-gentiobioside	3,082,301	550.5	29307-60-6
Gardoside	46,173,850	374.34	54835-76-6
Shanzhiside	11,948,668	392.35	29836-27-9

### 2.2. Screening of potential targets for HE

We used online tool DisGeNET to find potential therapeutic targets for HE. DisGeNET is a discovery platform containing 1 of the largest publicly available collections of genes and variants associated to human diseases.^[30–32]^ By searching the key word “HE,” “Minimal HE” and “Chronic hepatic encephalopathy,” then selecting the “ Evidences for Gene-Disease Associations “ option, the targets related to HE were collected.

### 2.3. Screening of potential therapeutic targets of IGFG for HE

The targets information including gene ID, name, and organism was identified using UniProt database (https://www.uniprot.org/).^[33]^ Then, common targets of IGFG and HE were identified through Venn analysis to obtain potential targets for IGFG in treating HE.

### 2.4. Construction of chemicals-target-disease network

In order to elucidate the relationship among IGFG, HE and their related targets, a “chemicals-target-disease (C-T-D)” network was constructed through Cytoscape 3.7.2 (http://www.cytoscape.org/). Cytoscape 3.7.2 is an open-source software platform which is used for visualizing complicated biomolecular networks and integrating different types of attribute data.^[34]^ In the network, nodes represent IGFG, HE and their related targets, while the connections between the nodes represent the interactions between these biological analyses.

### 2.5. Construction of PPI network and screening of core targets

In this study, Search Tool for the STRING (https://string-db.org/)^[35]^ was used to collect possible protein-protein interactions (PPI) by uploading the common targets that related to IGFG and HE. Species was limited to “Homo sapiens” with a confidence score > 0.4. Then, Cytoscape 3.7.2 software was used to construct the PPI networks and the degrees values of each target in the PPI network are obtained by analyzing plugin.^[36]^ The core targets are selected based on the degree values. The larger the value is, the more likely the target is the core target of IGFG in treating HE. In this study, the top eleven targets with the largest degree were screened as core targets.

### 2.6. GO and KEGG analysis

For the eleven potential core targets of IGFG anti-HE obtained from PPI analysis in the previous step, the OmicShare (http://www.omicshare.com/tools) was applied to perform gene ontology (GO)^[37]^ and KEGG enrichment analysis.^[38]^ The enrichment analysis were all performed by “Homo” sapiens, and enrichment results with *P*<.01 were screened out to obtain enrichment and closely related biological processes (BPs) and signaling pathways. GO annotates genes and protein functions into 3 main items: BP, molecular function (MF), and cellular component (CC).

### 2.7. Construction of chemicals-target-pathway network

For the main signaling pathways obtained from the enrichment analysis in the previous step along with corresponding core targets and active ingredients, an illustrated network containing main “chemical target pathway (C-T-P)” was constructed to predict the potential key components of IGFG in treatment of HE. In this network, nodes of different colors and shapes represented the pharmacodynamic components, potential core targets, and main action pathways. Edge was used to connect a certain pharmacodynamic component with its potential target and the relevant pathways of the target. The degree value represents the number of other nodes connected to the node in the network.^[39]^ In this study, the compound with the highest degree value was predicted as the key components of IGFG in treatment of HE and used for molecular docking validation with core targets.

### 2.8. Molecular docking

Molecular docking is used to validate the interactions between key compounds and core targets in network pharmacology analysis results. Download the 2-dimensional (2D) structure of the key compounds from PubChem (https://pubchem.ncbi.nlm.nih.gov/) and the 3-dimensional (3D) structure of the core targets from RCSB PDB (https://www.rcsb.org/).^[40]^ The protein structure was optimized by energy minimization by incorporating Tripos force field.^[41]^ SYBYL-X 2.1.1 software is used for molecular docking. The blot value is set to 1.0 and the threshold is set to 0.5.^[42,43]^ The stable conformation of low energy between ligand and receptor indicates that there is a great possibility of interaction between them. Generally, binding energy ≤ –5 kJ/ mol is used as the screening standard. In this Surflex dock, The best docked ligand pose was identified by considering the Total Score (T-score), which is converted into the binding free energy (formula Δ G0 = –2.303 RT × total score, where R is the ideal gas constant of molecule and t is the thermodynamic temperature of ideal gas).

## 3. Results

### 3.1. Potential therapeutic targets of IGFG for HE

**Figure 2.**
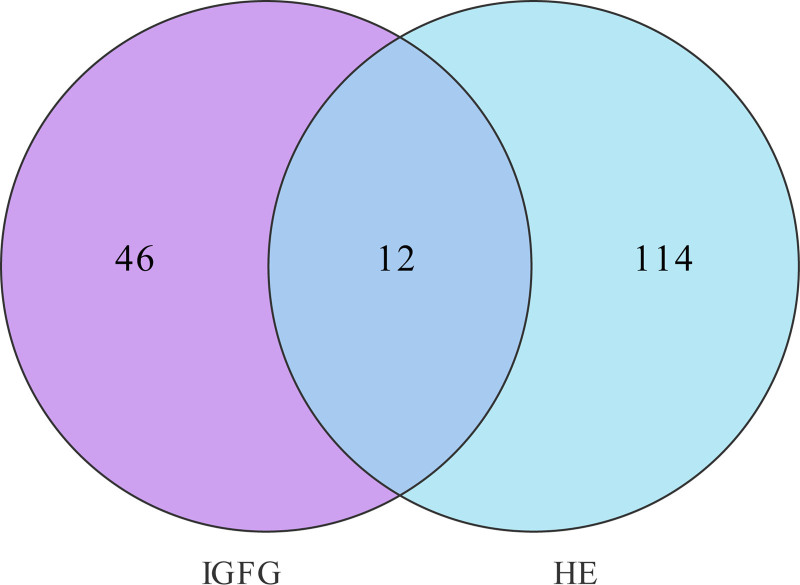
Venn analysis diagram of IGFG and HE. Purple section represent the potential targets of IGFG and cyan section represent the potential targets of HE. HE = hepatic encephalopathy, IGFG = iridoid glycosides from *Fructus Gardeniae*.

Through the afore mentioned target acquisition methods, a total of 53 targets interacted with IGFG and 126 targets interacted with HE. The common targets of IGFG and HE were obtained by Venn analysis. The results showed that there were 12 common targets of IGFG in treatment with HE. The Venn analysis diagram is shown in Figure 2.

### 3.2. Chemicals-target-disease network construction and analysis

**Figure 3.**
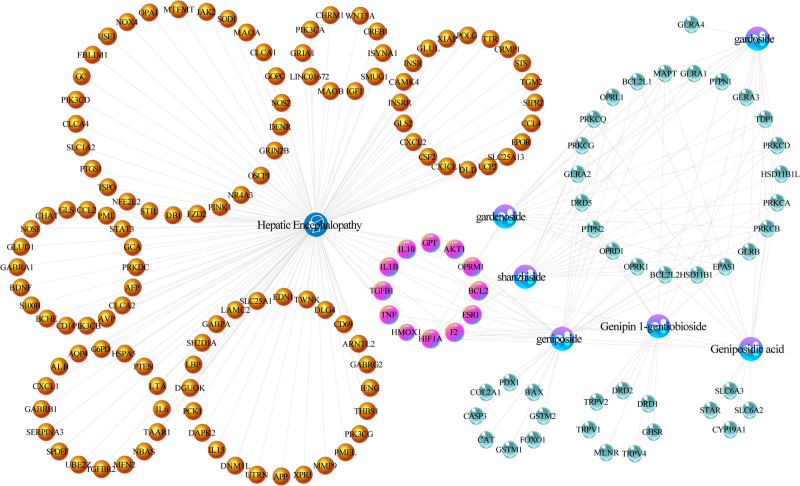
The chemicals-target-disease network generated in this study. the purple capsule-shaped nodes represent IGFG, the blue head-shaped nodes represent the diseases (HE), the yellow nodes, cyan and pink nodes represent the potential targets, while the edges represent the interactions between nodes. HE = hepatic encephalopathy, IGFG = iridoid glycosides from *Fructus Gardeniae*.

In order to more intuitively present the network topology status of IGFG in treatment of HE, “chemicals-target-disease (C-T-D)” network was constructed. The purple nodes represent IGFG, the blue nodules represent HE, and the pink nodes represent 12 common targets between IGFG and HE In the network, which are AKT1, BCL2, ESR1, F2, GPT, HIF1A, IL10, IL1B, OPRM1, TGFB1, Tumor necrosis factor (TNF), and HMOX1. Then the yellow nodes represent other non-intersection related target of HE, the cyan target clusters are other non-intersection related target of IGFG. The chemicals-target-disease network is shown in Figure 3. Relevant data see Tables S1, Supplemental Digital Content, http://links.lww.com/MD/O249 and S2, Supplemental Digital Content, http://links.lww.com/MD/O249, which lists the detailed raw data of C-T-D network related relationships.

### 3.3. PPI network construction and core targets

**Figure 4.**
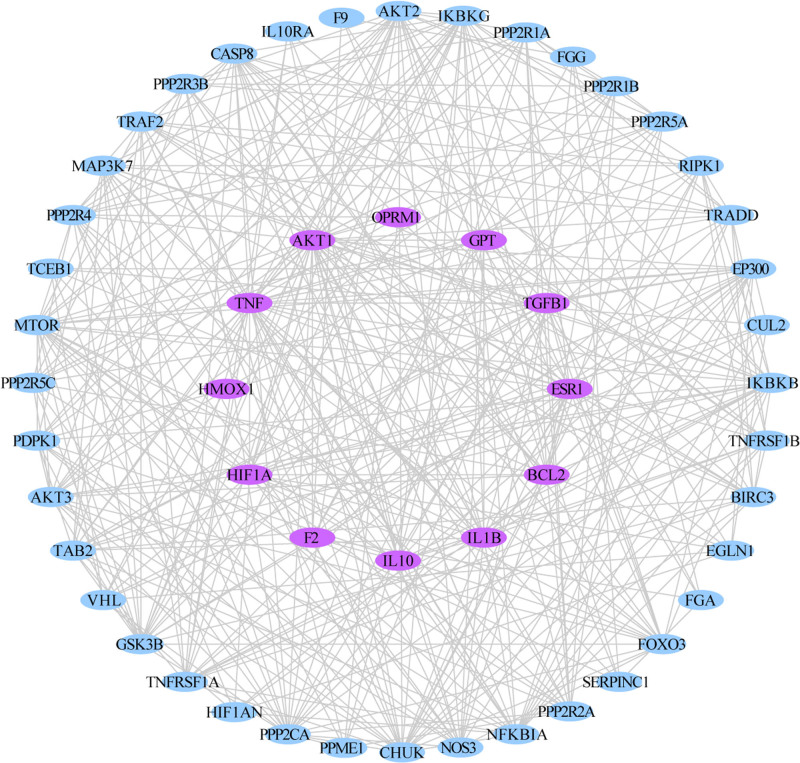
The PPI network generated in this study. the pink nodes represent the 11 common targets, the blue nodes represent the related active proteins obtained by the STRING Tool, while the edges represent the interactions between nodes.

PPI network was constructed from the 12 common targets of IGFG and HE. It contains 52 nodes and 397 edges. The purple nodes represent common targets, the blue nodes represent other targets. PPI network topology analysis results: network heterogeneity is 0.452, network density is 0.308, the average betweenness centrality of nodes is 0.0196, the shortest path is 2430 and the average degrees 16.2447. The PPI network is shown in Figure 4. Relevant data see Table S3, Supplemental Digital Content, http://links.lww.com/MD/O249 Supplemental Content, which lists the detailed raw data of PPI network related relationships.

**Figure 5.**
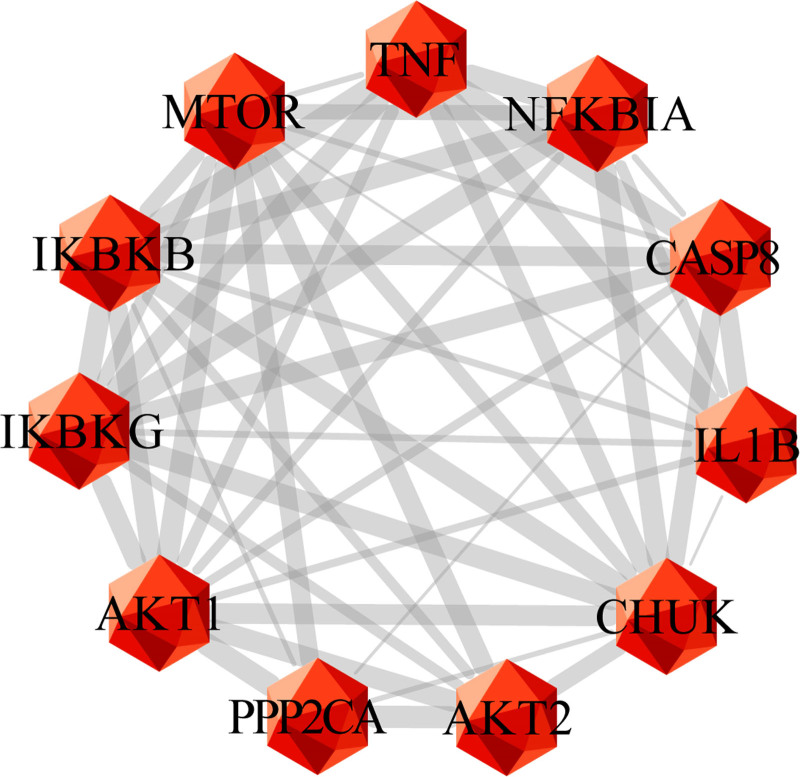
The core targets network generated in this study. the red nodes represent the core targets, the edges represent the interactions between nodes.

By conducting topological analysis on the PPI network, AKT1, TNF, MTOR, CHUK, PPP2CA, IKBKB, AKT2, IKBKG, IL1B, NFKBIA, and CASP8 are predicted to be the core targets of IGFG in treating HE (degree > 20). In this study, the combined scores of 11 core targets were obtained from string database, and then the core targets and combined scores were imported into Cytoscape software to construct the core target network. In the network, the red nodes represent the core targets, the edges represent the interactions between nodes, while the edge thickness varies according to the combined score. The core target network is shown in Figure 5.

### 3.4. Analysis of GO enrichment

**Figure 6.**
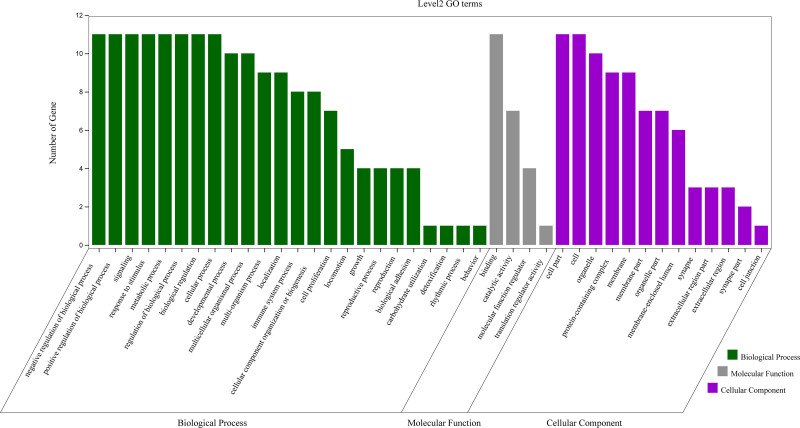
The second level GO functional enrichment analysis statistics of core targets. The vertical axis represents the number of gene, the horizontal axis represents the level 2 GO terms, the green bars represent biological process, the gray bars represent molecular function, the purple bars represent cellular component. GO = gene ontology.

**Figure 7.**
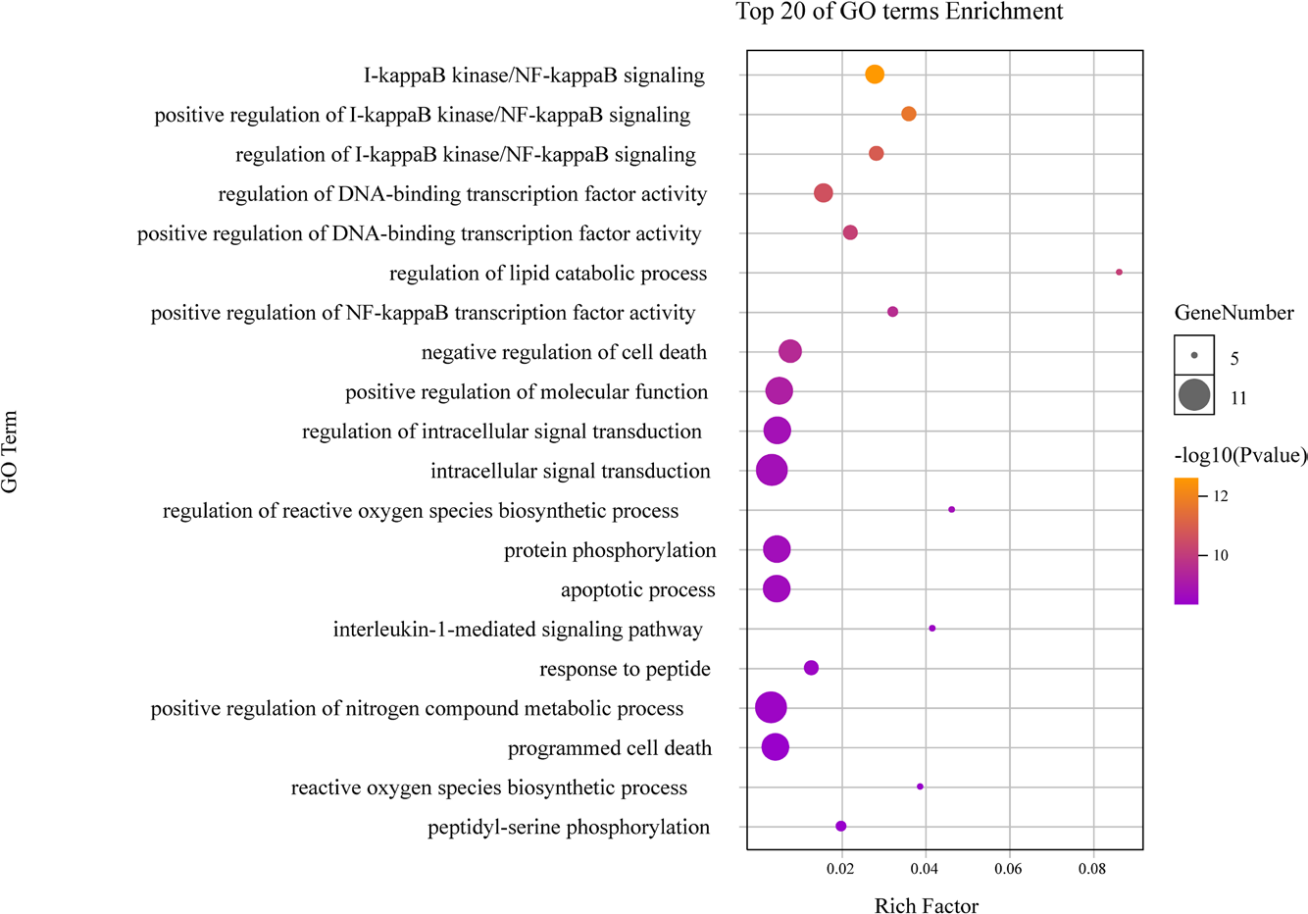
The top 20 GO enrichments in BP. The vertical axis represents the GO term name, the horizontal axis represents the rich factor, the size of the dot indicates the number of genes expressed in the GO term, and the color of the dot corresponds to the different *P*-value range. BP = biological process, GO = gene ontology.

**Figure 8.**
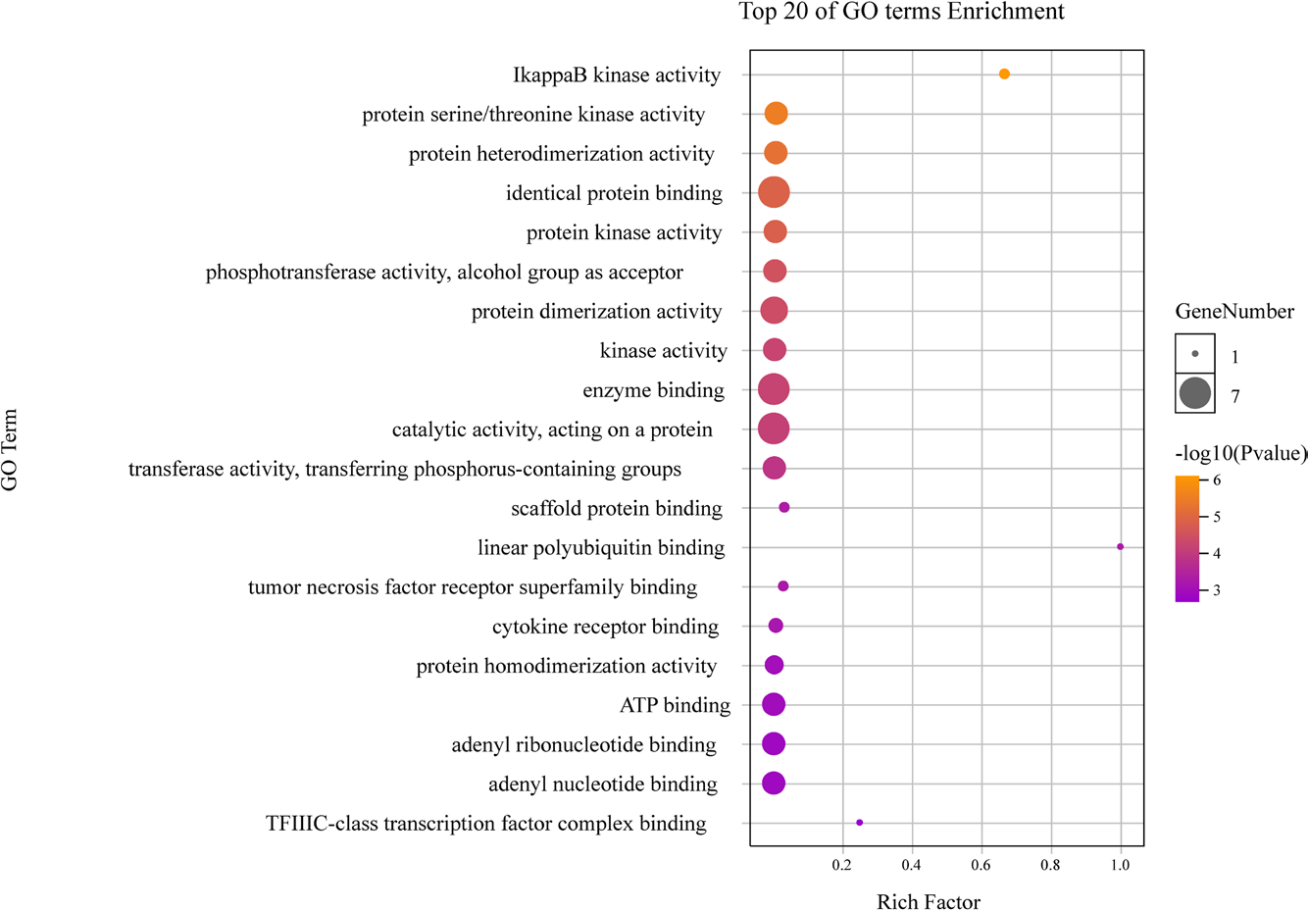
The top 20 GO enrichments in MF. The vertical axis represents the GO term name, the horizontal axis represents the rich factor, the size of the dot indicates the number of genes expressed in the GO term, and the color of the dot corresponds to the different *P*-value range. GO = gene ontology, MF = molecular function.

**Figure 9.**
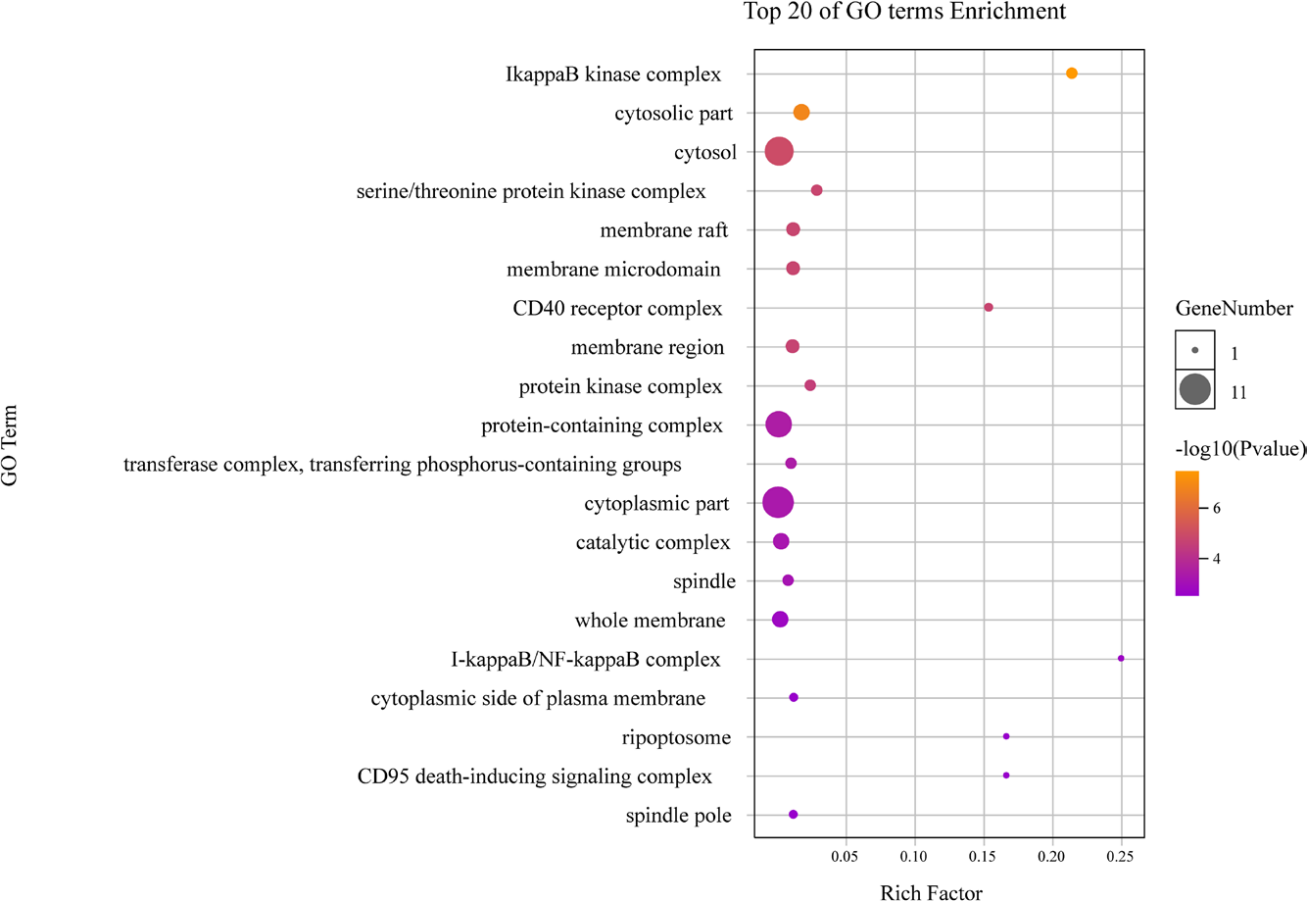
The top 20 GO enrichments in CC. The vertical axis represents the GO term name, the horizontal axis represents the rich factor, the size of the dot indicates the number of genes expressed in the GO term, and the color of the dot corresponds to the different *P*-value range. CC = cellular component, GO = gene ontology.

As highlighted in Figure 6, different categories of BP, MF, and CC were represented by a green, gray and purple bar, respectively. The height of the bar represented the number of genes observed in the category. Each *P*-value of enrichment results was calculated and *P*-value < .01 was considered to be significantly enriched. Ranking p-values according to the order from small to large, the top 20 BP, MF, CC terms are displayed in bubble charts as shown in Figures 7 to 9, respectively. There are 1196 enriched entries related to BP which cover I-kappaB kinase/NF-kappaB signaling, regulation of DNA-binding transcription factor activity, regulation of lipid catabolic process, positive regulation of NF-kappaB transcription factor activity, negative regulation of cell death, positive regulation of MF; 49 enriched entries related to MF, which includes I-kappaB kinase activity, protein serine/threonine kinase activity, protein heterodimerization activity, identical protein binding, protein kinase activity; 35 enriched entries related to CC, involving I-kappaB kinase complex, serine/threonine protein kinase complex, membrane microdomain, membrane raft, cytosolic part and cytosol, etc.

### 3.5. Analysis of KEGG pathway enrichment

**Figure 10.**
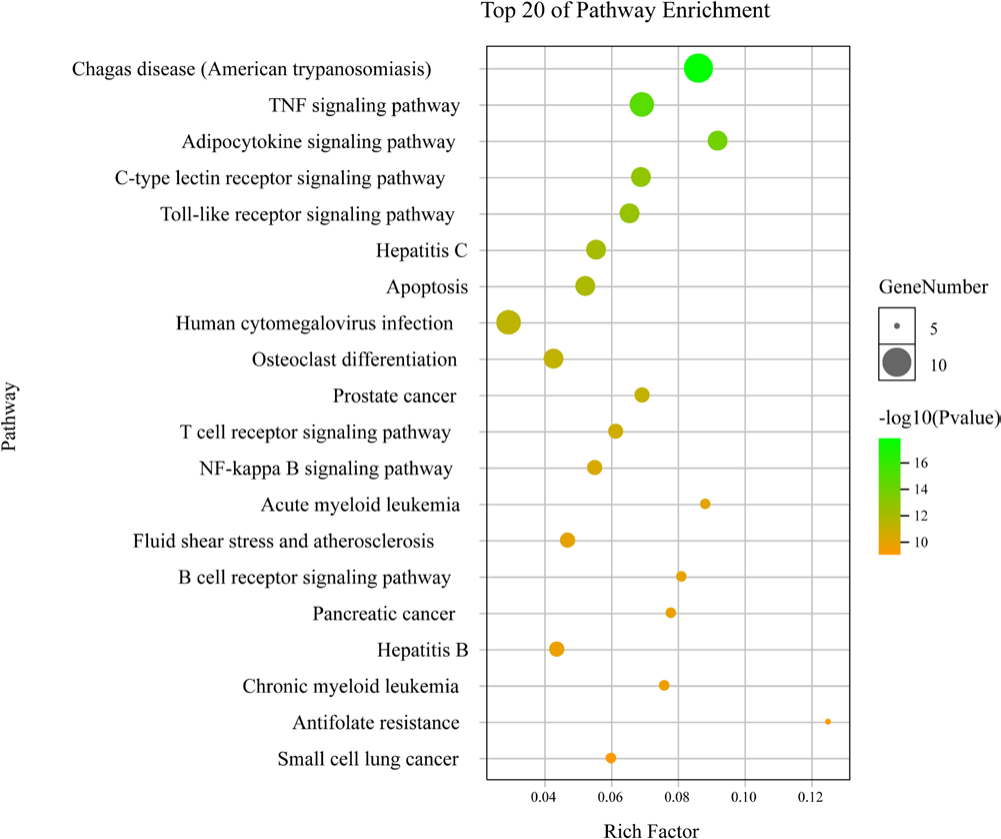
Pathway enrichment point diagram of core targets. The vertical axis represents the pathway name, the horizontal axis represents the rich factor, the size of the dot indicates the number of genes expressed in the pathway, and the color of the dot corresponds to the different *P*-value range.

To further reveal the potential therapeutic mechanism of IGFG on HE, we conducted KEGG pathway enrichment analysis. The result showed that 11 core targets were enriched into 132 signaling pathways, of which 98 pathways showing significant correlation (*P* < .01). The top 20 pathways with lower *P*-values are listed in Table 2, including Chagas disease (American trypanosomiasis), TNF signaling pathway, adipocytokine signaling pathway, C-type lectin receptor signaling pathway, toll-like receptor (TLR) signaling pathway, NF-kappa B signaling pathway, osteoclast differentiation, prostate cancer, t cell receptor signaling pathway, hepatitis c, apoptosis and human cytomegalovirus infection, etc. These pathways mainly involve infectious diseases, signal transduction, endocrine system, immune system, cell growth and death, development, cancers, cardiovascular diseases and drug resistance. The top 20 enrichment results of KEGG are shown in Figure 10. The size of nodes in figure depends on the number of related targets, and the more targets there are, the larger the nodes will be; The color of the node depends on the *P*-value, and the color from yellow to green reflects the *P*-value from high to low.

**Table 2:** Classification and information on core targets-related pathways, direct (top 20).

Level 1 classification	Level 2 classification	Pathway	*P*-value	Genes count
Human diseases	Infectious diseases	Chagas disease (American trypanosomiasis)	1.617e−18	10
Environmental information processing	Signal transduction	TNF signaling pathway	1.82944e−15	9
Organismal systems	Endocrine system	Adipocytokine signaling pathway	1.37793e−14	8
Organismal systems	Immune system	C-type lectin receptor signaling pathway	1.48441e−13	8
Organismal systems	Immune system	Toll-like receptor signaling pathway	2.24557e−13	8
Human diseases	Infectious diseases	Hepatitis C	8.71252e−13	8
Cellular processes	Cell growth and death	Apoptosis	1.42782e−12	8
Human diseases	Infectious diseases	Human cytomegalovirus infection	4.74253e−12	9
Organismal systems	Development	Osteoclast differentiation	7.27884e−12	8
Human diseases	Cancers	Prostate cancer	8.50595e−12	7
Organismal systems	Immune system	T cell receptor signaling pathway	2.02393e−11	7
Environmental information processing	Signal transduction	NF-kappa B signaling pathway	4.37154e−11	7
Human diseases	Cancers	Acute myeloid leukemia	9.28029e−11	6
Human diseases	Cardiovascular diseases	Fluid shear stress and atherosclerosis	1.35919e−10	7
Organismal systems	Immune system	B cell receptor signaling pathway	1.56593e−10	6
Human diseases	Cancers	Pancreatic cancer	2.00111e−10	6
Human diseases	Infectious diseases	Hepatitis B	2.24984e−10	7
Human diseases	Cancers	Chronic myeloid leukemia	2.34367e−10	6
Human diseases	Drug resistance	Antifolate resistance	7.9974e−10	5
Human diseases	Cancers	Small cell lung cancer	9.94432e−10	6

### 3.6. Chemicals-target-pathway network construction and analysis

**Figure 11.**
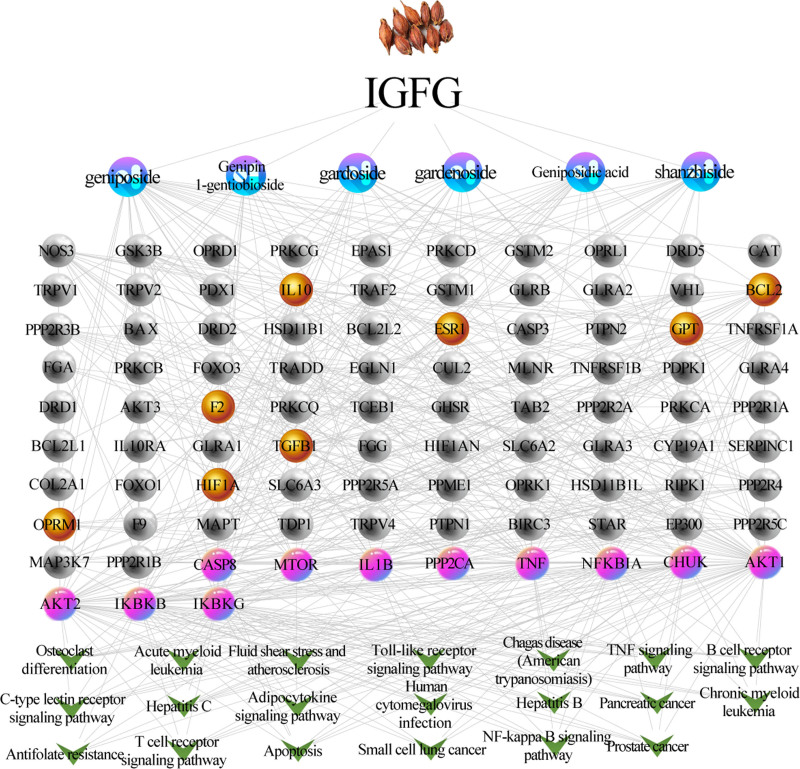
The chemicals-target-pathway network generated in this study. the purple capsule-shaped nodes represent the candidate compounds in IGFG; the yellow, pink and gray nodes represent putative targets of IGFG. Among them, the yellow nodes represent the common targets of IGFG and HE, the pink nodes represent core targets of IGFG for the treatment of HE and the green V nodes represent the main pathways (top 20) of core targets. HE = hepatic encephalopathy, IGFG = iridoid glycosides from *Fructus Gardeniae*.

Using the merge function, a chemicals-target-pathway (C-T-P) network was constructed with 120 nodes (1 node represents IGFG, 93 potential targets, 6 candidate components and 20 related pathways) and 648 edges. As shown in Figure 11, the purple capsule-shaped nodes represent the candidate compounds in IGFG; the yellow, pink and gray nodes represent putative targets of IGFG. Among them, the yellow nodes represent the common targets of IGFG and HE, the pink nodes represent core targets of IGFG for the treatment of HE and the green V nodes represent the main pathways (top 20) of core targets; while the edges represent the interactions between nodes. Results of the network topology analysis are as follows: network density (0.091), network heterogeneity (1.019), and shortest paths (14,280, 100%). The average degree of nodes is 10.8, and there are 45 nodes larger than the average degree. The average betweenness centrality of nodes is 0.01413, and there are 23 nodes larger than the average betweenness centrality. Moreover, the degree value of active components was analyzed based on the topological properties. Here, geniposide with highest degree value (30) was screened as key active component for molecular docking verification with the 11 core targets. Relevant data see Table S4, Supplemental Digital Content, http://links.lww.com/MD/O249, which lists the detailed raw data of C-T-P network related relationships.

### 3.7. Results of molecular docking

**Figure 12.**
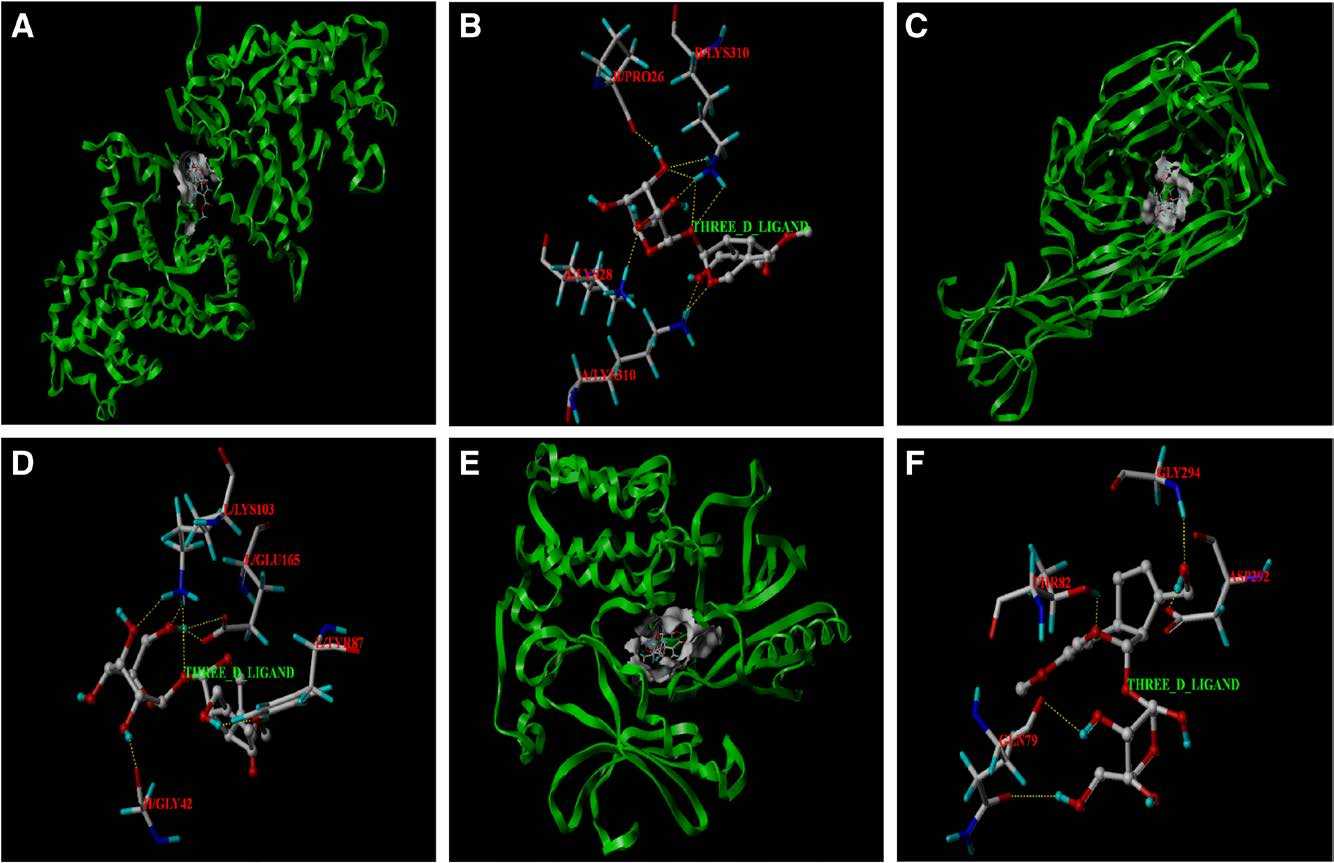
Molecular docking models of geniposide binding to the 6 core targets. (A) Geniposide was docked to the surface of PPP2CA. (B) The binding interaction between geniposide and PPP2CA protein. (C) Geniposide was docked to the surface of TNF. (D) The binding interaction between geniposide and TNF protein. (E) Geniposide was docked to the surface of AKT1. (F) The binding interaction between geniposide and AKT1 protein. TNF = tumor necrosis factor.

The structure of core targets and key compound with highest degree value were introduced into SYBYL-X 2.1.1 software for molecular docking. In this paper, we observed that geniposide can enter into the active pockets of 11 core targets protein respectively. The results of molecular docking analysis showed that the binding free energy (∆G in kcal/mol) value of geniposide to the core target was negative, indicating that the ligand molecule could spontaneously bind to the receptor protein. Furthermore, the binding energy in this result was less than −5 kJ/ mol, which further proved the strong binding ability. The binding energies of the geniposide with various core targets are shown in Table 3. In general, the lower the binding free energy, the more stable the binding between the ligand and protein receptor. According to the results of molecular docking, the binding free energies of PPP2CA, TNF and AKT1 with geniposide are the lowest, as shown in Figure 12.

**Table 3:** The binding energy of the geniposide with various core targets.

Compound	Core target	PDB ID	T-score	Binding energy (kJ/mol)
Geniposide	PPP2CA	4NY3	8.6573	–49.42210501
Geniposide	TNF	3ALQ	6.8107	–38.88038194
Geniposide	AKT1	6HHF	6.6161	–37.76946495
Geniposide	NFKBIA	1NFI	5.9039	–33.70371429
Geniposide	CHUK	5TQW	5.7473	–32.80972868
Geniposide	AKT2	3E8D	5.6566	–32.29194774
Geniposide	MTOR	3JBZ	5.5816	–31.86379371
Geniposide	IKBKB	4KIK	5.2401	–29.91426570
Geniposide	CASP8	3KJN	5.0725	–28.95748416
Geniposide	IKBKG	3FX0	4.3280	–24.70734184
Geniposide	IL1B	6Y8M	4.0190	–22.94334724

## 4. Discussion

Hepatic encephalopathy is a serious neuropsychiatric complication of cirrhosis or porto-systemic shunting. Its clinical symptoms vary greatly, extending from subtle impairment in mental state to coma or even death, accompanied by cognitive impairment, attention deficits, motor incoordination, and psychomotor slowing, which impair quality of life, and increase accidents and hospitalizations, so as to reduce the lifespan. HE affects several million people and is a serious health, social, and economic problem.^[7,44]^ TCM has multi-component and multi-target characteristics, and it has its unique advantages in treating complex diseases with the concept of “holistic treatment.” Network pharmacology is the discovery of TCM from the system and molecular level. The past research model of “one target, 1 drug” is updated to the new “network target, multi-component” model. It can effectively predict the targeted distribution and pharmacological effects of TCM compounds by co-expression analysing disease-related genes.

This study suggests that there are 12 common targets between IGFG and HE. However, proteins in the human body do not exist independently and there are complex interactions between them. Therefore, we construct the PPI network to predict PPI in order to determine functional connections between target proteins, and ultimately identified 11 core targets based on degree values, namely AKT1, TNF, MTOR, CHUK, PPP2CA, IKBKB, AKT2, IKBKG, IL1B, NFKBIA, and CASP8.

In order to explore the multi-dimensional pharmacological mechanism of IGFG on HE, GO and KEGG enrichment were conducted in this study. We analyzed the pathways with higher enrichment levels. Firstly, Hepatitis C, Hepatitis B and Human cytomegalovirus infection were mainly related to viral infectious diseases. Based on this, we infer that IGFG may have broad-spectrum antiviral effects, especially against hepatitis viruses; Secondly, TNF signaling pathway and NF-κB signaling pathway are the 2 pathways that we are most concerned about, which are closely related to peripheral inflammation and neuroinflammation. Studies have shown that hyperammonemia and peripheral inflammation play synergistic roles in inducing cognitive and motor alterations in HE.^[45]^ The emergence of neurological alterations is mediated by the induction of neuroinflammation, which alters glutamatergic and GABAergic neurotransmission, leading to cognitive and motor disorders.^[46,47]^ TNF, as a critical cytokine, can induce a wide range of intracellular signal pathways including apoptosis and cell survival as well as inflammation and immunity. In the central nervous system, microglia and astrocytes are the main sources of TNF-a. TNF-a expression is also induced in neurons in the hippocampi of rats with HE.^[48]^ NF-κB is a major transcription factor that controls the expression of many proinflammatory genes, including TNF-a,^[49,50]^ and participates in inflammasome regulation.^[51]^ Studies have shown that TNF-a produced in glia activates TNFR1 in Purkinje neurons, inducing the translocation of NF-κB to the nucleus and increasing the transcription of TNF-a mRNA and the synthesis of the TNF-a protein. TNF-α causes an autocrine loop both in glia and neurons, further exacerbating the effects of neuroinflammation in the brain^[44]^; Thirdly, C-type lectin receptor signaling pathway, TLR signaling pathway, T cell receptor signaling pathway and B cell receptor signaling pathway are related to the immune system and are also pathways of greater concern to us. Studies have shown that the emergence of HE is due to the changes in peripheral immune system, which are transmitted to brain, leading to neuroinflammation that alters neurotransmission leading to cognitive and motor alterations.^[52]^ Especially, TLR is an important component of the innate immune system. Studies have found that the expression of TLR9 serves as a biomarker that determines presence and severity of encephalopathy in acute liver failure and cirrhosis, which can be used as a useful biomarker that differentiates those who develop high grade HE from those who do not^[53]^; Furthermore, Adipocytokine signaling pathway and Apoptosis are also worthy of attention. Adipocytokines are mainly bioactive substances produced by adipocytes, which play an important role in the development of steatosis, inflammatory necrosis, fibrosis and cirrhosis of liver.^[54]^ For Apoptosis, studies have shown that changes in the expression levels of cyclin A and D1, cell cycle arrest, and apoptosis may cause hyperammonemia induced liver injury in rats.^[55]^ Regulation of these 2 pathways can alleviate the deterioration of liver disease and prevent the occurrence of HE.

This study predicts that geniposide is the key active component with highest degree value of IGFG in treatment of HE, based on the construction and topology analysis of the chemical target pathway (C-T-P) network model. Then molecular docking was utilized to validate the binding affinity between 11 core targets and geniposide. Molecular docking is a prevalent strategy for assessing molecule-target interactions. The results showed that the binding free energy of geniposide with the all 11 core target was much lower than −5 kJ/mol, indicating that the ligand molecule could spontaneously bind to the receptor protein and had strong binding force, which verifies the reliability of network pharmacology analysis from a mechanical perspective. Our data demonstrate that the effectiveness and mechanism of IGFG in treating HE are complex, involving interactions between multiple components, and among which geniposide is a potential quality marker for IGFG in treating HE. This may provide new insights into the development of novel therapeutics for HE.

Our study only employed solely up-to-date bioinformatics methods to initiate an exposition of the mechanism of IGFG in treating HE from the theoretical level using network pharmacology and molecular docking. Therefore, further experimental validation in vivo and in vitro and clinical trials are needed for future research. Nevertheless, the outcomes of this study offer commendable directions for the design of our experimental undertaking.

## 5. Conclusion

This study utilized a combination of network pharmacology and molecular docking to investigate the potential mechanism of IGFG therapy for HE. IGFG can inhibit inflammatory reaction, regulate immunity, promote hepatocyte regeneration, reduce hepatocyte apoptosis, maintain liver function homeostasis and antiviral function through direct targeting and signaling pathway regulation, which reflects the characteristics of IGFG overall regulation and network regulation. This can provide direction for further study on key targets, biological effects and molecular mechanism of IGFG in the treatment of HE, and provide scientific basis for clinical treatment of HE.

## Author contributions

**Conceptualization:** Fangzhou Liu, Meng Li, Yuanbai Li, Yihao Li, Yang Yang.

**Data curation:** Fangzhou Liu, Meng Li, Yuanbai Li, Yihao Li, Yang Yang.

**Formal analysis:** Fangzhou Liu, Meng Li, Yuanbai Li, Yu Du, Yihao Li, Yang Yang.

**Funding acquisition:** Fangzhou Liu, Yuanbai Li, Yang Yang.

**Investigation:** Fangzhou Liu, Yu Du, Yihao Li, Yang Yang.

**Methodology:** Fangzhou Liu, Meng Li, Yihao Li, Yang Yang.

**Project administration:** Fangzhou Liu, Yang Yang.

**Resources:** Fangzhou Liu, Yang Yang.

**Software:** Fangzhou Liu, Yuanbai Li, Yu Du, Yang Yang.

**Supervision:** Fangzhou Liu, Yuanbai Li, Yang Yang.

**Validation:** Yu Du, Yihao Li, Yang Yang.

**Visualization:** Fangzhou Liu, Meng Li, Yuanbai Li, Yu Du, Yang Yang.

**Writing – original draft:** Fangzhou Liu.

**Writing – review & editing:** Yihao Li, Yang Yang.

## Supplementary Material


